# Prevalence and associated factors of apathy in Chinese ALS patients

**DOI:** 10.3389/fpsyg.2023.1089856

**Published:** 2023-03-30

**Authors:** Qian-Qian Wei, Yuan Guo, Shirong Li, Tianmi Yang, Yanbing Hou, Ruwei Ou, Junyu Lin, Qirui Jiang, Huifang Shang

**Affiliations:** ^1^Laboratory of Neurodegenerative Disorders, Department of Neurology, West China Hospital, Sichuan University, Chengdu, Sichuan, China; ^2^Outpatient Department, West China School of Nursing, West China Hospital, Sichuan University, Chengdu, Sichuan, China; ^3^Department of Neurology, Guizhou Provincial People’s Hospital, Guiyang, Guizhou, China

**Keywords:** amyotrophic lateral sclerosis, apathy, depression, anxiety, health-related quality of life, survival

## Abstract

**Objectivve:**

This study aimed to explore the prevalence and clinical correlates of apathy in amyotrophic lateral sclerosis (ALS) in a cohort of Chinese patients.

**Methods:**

A total of 1,013 ALS patients were enrolled in this study. Apathy was recorded during face-to-face interviews using Frontal Behavioral Inventory, and other patient characteristics, including depression, anxiety, and cognitive function, were collected using Hamilton Depression Rating Scale (HDRS), Hamilton Anxiety Rating Scale (HARS), and Chinese version of Addenbrooke’s Cognitive Examination-revised. Health-related quality of life of ALS patients and their caregivers was also evaluated, and the potential factors associated with apathy were explored using forward binary regression analysis. Survival was analyzed using the Cox proportional hazards model.

**Results:**

The prevalence of apathy in all patients was 28.9%. Patients in the late disease stage had a higher prevalence of apathy than those in the early disease stage. Furthermore, patients with apathy had a lower ALS Functional Rating Scale revised (ALSFRS-R) score, higher HDRS score, HARS score and higher proportion of reported problems in the anxiety/depression. Additionally, their caregivers had higher score of depression and higher Zarit-Burden Interview scores. Multivariate regression analysis revealed that apathy in ALS was associated with the onset region (*p* = 0.027), ALSFRS-R score (*p* = 0.007), depression (*p* = 0.001) and anxiety (*p* < 0.001). Apathy had a significant negative effect on survival in ALS patients (*p* = 0.032).

**Conclusion:**

Apathy is relatively common (28.9%) in Chinese patients with ALS. Apathy is related to both the severity of the disease, and the presentation of non-motor symptoms in ALS, such as depression and anxiety disorders. Apathy is an independent prognostic factor for survival and requires early intervention and management.

## Introduction

Amyotrophic lateral sclerosis (ALS) is an irreversible progressive motor neurodegenerative disease featured with a deterioration of the upper and/or lower motor neurons (Brown and Al-Chalabi, 2017). In addition to the typical clinical symptoms of limb weakness, dysarthria, dysphagia, and muscle atrophy, ALS patients also experience a profile of cognitive impairments and behavioral changes in different degree. Cognitive changes are characterized by executive and memory dysfunction, broadening to impairments in social cognition and language (Goldstein and Abrahams, 2013). Behavioral changes in ALS manifests most commonly as apathy, loss of sympathy, disinhibition and changes in eating habits (Pender et al., 2020).

Among the many neuropsychiatric changes associated with ALS; apathy has received the most scrutiny. Apathy is characterized by decreased motivation toward goal-directed behaviors, which are variably featured by reduced emotions or interests (Robert et al., 2018). It is one of the most prevalent neuropsychiatric symptoms in neurodegenerative diseases, such as behavioral variant frontotemporal dementia (FTD), Alzheimer’s disease (Leung et al., 2021), and Parkinson’s disease (Liu et al., 2017). The prevalence of apathy ranged from to 8.6 to 80% in ALS (Santangelo et al., 2017), depending on the use of variable assessment instruments and patients selected methods. The prevalence rate of apathy assessed by ALS patient informants or caregivers was significantly higher than that self-rated by the patients. One review showed that the frequency of apathy was 25% in studies that using self-rated tools and 34% in studies using informant-rated tools in ALS (Kutlubaev et al., 2022). This review study also found the lowest frequency of apathy in ALS was registered in the studies from Asia (Kutlubaev et al., 2022). Only a few studies were concerned about apathy in Chinese ALS. In our previous observational studies, apathy was a common impaired neurobehavioral domain with a prevalence of 16.5–19% in ALS patients, which is a lower rate than that reported in previous literature (Wei et al., 2014, 2016). Another study reported that 14% of patients displayed abnormal apathy behavior (Cui et al., 2015). Compared with German ALS patients, the frequency of apathy was lower in Chinese ALS patients, but the difference was not statistically significant (Ye et al., 2019). The occurrence and clinical correlates of apathy require further investigation.

In ALS, there is limited evidence to suggest associations between disease and non-motor features with apathy. Apathy is associated with bulbar involvement in ALS, but whether the bulbar onset or severity of bulbar symptoms contributes to the development of apathy remains unknown (Chiò et al., 2010). Previous studies reported that there was no association between the apathy score and ALS Functional Rating Scale revised (ALSFRS-R) score (Chiò et al., 2010; Salas et al., 2020). Further, there was no consistent correlation between apathy and depression severity or quality of life (QoL). An observational study with a small simple size found that patients with apathy showed higher levels of depression and lower QoL than non-apathetic patients (Caga et al., 2018b); however, no association was found in other studies (Bock et al., 2016; Caga et al., 2016; Radakovic et al., 2017). The development of apathy has been related to cognitive decline in one study (Unglik et al., 2018), while another showed that patients with cognitively impaired ALS have worse apathy scores (Witgert et al., 2010). Apathy may be a critical prognostic factor in ALS. A longitudinal study of 76 patients with ALS indicated that apathy was a significant predictor of survival after adjusting for cognitive status and other clinical parameters (Caga et al., 2016). A study of 152 patients indicated that apathy negatively correlated with survival time (Unglik et al., 2018). Some studies have found no association between apathy scores and survival (Mioshi et al., 2014, 2015). Another observational study of 51 patients found that apathy was related to a higher care burden in ALS caregivers (Caga et al., 2018a). Apathy may even affect the sufferer’s ability to engage competently in end-of-life decisions. Therefore, a better understanding of apathy would be helpful in personalized care and early intervention.

Therefore, the study was designed to examine the prevalence and clinical correlates (emotional state and cognitive function) of apathy in ALS patients. We further investigated the associations between apathy and QOL in ALS and care burden in their caregivers. Finally, we established the influence of apathy on ALS prognosis.

## Patients and methods

The study was conducted in our motor neuron disease center from southwest China from August 2012 to July 2021. According to the revised El Escorial criteria, definite or probable ALS were enrolled. Written informed consent was obtained from all participants. The study was approved by the Ethics Committee of the West China Hospital of Sichuan University [approval No.2015 (236)].

Demographic data and disease-related data were collected. The ALS Functional Rating Scale revised (ALSFRS-R) scale was used to evaluate functional impairment. The rate of disease progression was evaluated as the changes in ALSFRS-R per month (Formula: (48-ALSFRS-R score at the baseline)/month intervals between first symptom onset and baseline). The disease stage was identified using the King’s College Staging System. Early stage subgroup included patients from stage 1 or stage 2 and late-stage subgroup included patients from stage 3 or stage 4.

The Frontal Assessment Battery (FAB) to assess frontal lobe executive function in a face-to-face interview. A Hamilton Depression Rating Scale-24 (HDRS) score > 20 indicates depression and a Hamilton Anxiety Rating Scale (HARS) score > 14 indicates anxiety. Cognitive dysfunction was defined as score of less than 75 in our previous study using the Chinese version of Addenbrooke’s Cognitive Examination-revised (ACE-R) (Wei et al., 2015). Frontal Behavioral Inventory (FBI) was used to frontal behavioural symptoms. Lower scores indicate better frontal behavioral function. Item number one of the FBI was used to evaluate apathy in ALS patients. Patients with scores ≥1 were considered to be “with the presence of apathy,” <1 as “without the presence of apathy.” Health-related QoL was assessed using the five-level EuroQol five-dimension (EQ-5D-5L) scale, which is a standardized QoL scale. The basic activities of daily living (BADL) and instrumental activities of daily living (IADL) were used to evaluate QoL and ALS ability. Higher scores indicate a better QoL. Caregivers’ depressive symptoms and burden were investigated by Beck Depression Inventory (BDI) and Zarit-Burden Interview (ZBI). All clinical and treatment data, including ALSFRS-R score, medication, survival and other treatment, were collected in the followed-up.

## Statistical analysis

All analyses were performed using SPSS 26.0 (SPSS, Inc., Chicago, IL, USA). Continuous parameters that were normally distributed are described as mean ± standard deviation (SD), and those with a non-normal distribution are presented as median values. Categorical variables are presented as percentages. Comparisons between the groups were performed using Student’s t-test or Chi-square test. The Bonferroni correction were applied in multiple comparisons. Stepwise backward binary logistic regression analysis was used to assess the potential factors associated with the presence of apathy. Kaplan–Meier (KM) curves and log-rank tests were used to assess survival in univariate analysis. The censoring time for follow-up was the end of April 2022. Multivariate analysis was performed using the Cox proportional hazards regression model to assess the effect of apathy on survival (stepwise forward). Hazard ratios (HRs) and 95% confidence interval (CI) were also calculated. A level of *p* < 0.05 was statistically significant.

## Results

The demographic and clinical features of the ALS patients are shown in Table 1. The prevalence of apathy among all registered patients was 28.9%. Intergroup comparisons showed that patients with apathy had significantly lower ALSFRS-R score than patients without apathy. While, the mean age, sex distribution, marital status, education level, body mass index (BMI), family history, disease duration, disease delay, onset region, and classical phenotype distribution did not differ in apathy subgroups.

**Table 1:** Demographic and clinical features of ALS patients.

Variables	Total sample	Without apathy	With apathy	*p*-value
Number	1,013	720	293	
Age (y)	54.4 ± 11.9	54.5 ± 12.0	54.2 ± 11.7	0.710
Onset age (y)	53.9 ± 11.0	54.0 ± 11.1	53.6 ± 10.9	0.594
Male, %	62.2	62.5	61.4	0.751
Married, %	95.6	95.8	94.9	0.505
Education (y)	9.2 ± 3.8	9.1 ± 3.9	9.3 ± 3.5	0.298
BMI	22.1 ± 3.0	22.1 ± 3.0	22.0 ± 3.0	0.517
Family history	1.6	1.4	2.0	0.446
Disease duration (m)	15.4 ± 14.0	15.5 ± 14.9	15.3 ± 11.8	0.863
Diagnostic delay (m)	14.2 ± 13.3	14.2 ± 14.0	13.9 ± 11.3	0.727
Onset region (bulbar, %)	16.5	16.0	17.7	0.562
Classical phenotype (%)	72.4	72.5	72.0	0.875
ALSFRS-R score	39.9 ± 5.4	40.3 ± 5.2	38.8 ± 5.7	<0.001*
ALSFRS-R bulbar	10.6 ± 1.8	10.7 ± 1.7	10.4 ± 1.9	0.044
ALSFRS-R Limb	17.6 ± 4.6	17.9 ± 4.5	16.9 ± 4.7	0.002*
ALSFRS-R Respiratory	11.7 ± 0.8	11.7 ± 0.8	11.6 ± 0.7	0.084
Disease progression rate	0.76 ± 0.69	0.73 ± 0.68	0.84 ± 0.71	0.024*

ALS, amyotrophic lateral sclerosis; ALSFRS-R, ALS functional rating scale revised; BMI, body mass index. *Significant difference. Student’s *t*-test and Chi-square test were used for between-group comparison. *p*-value represented the “*p*-value for between-group differences.”

The prevalence of apathy is related to ALS stage. Patients in the late-stage subgroup (Stage 3,34.3%; Stage 4,34.8%) had a higher percentage of apathy than patients in the early stage subgroup (Stage 1:21.9% and Stage 4:29.2%) (*p* = 0.009). The assessment of emotional state and cognitive function in ALS are listed in Table 2. Patients with apathy had significantly higher HDRS and HARS scores. Of the 293 patients with apathy, 201 (68.6%) reported depression and 157 (53.6%) reported anxiety. No differences in the FAB, ACE-R, and five domains of the ACE-R scores were found between apathy subgroups.

**Table 2:** Cognitive function and health-related quality of life of ALS patients in different subgroups.

Variables	Total sample	Without apathy	With apathy	*p*-value
HDRS	9.7 ± 7.3	8.4 ± 6.5	12.7 ± 8.2	<0.001*
Depression (%)	52.1	45.4	68.6	<0.001*
HARS	6.4 ± 5.8	5.4 ± 5.2	9.0 ± 6.5	<0.001*
Anxiety (%)	36.6	29.7	53.6	<0.001*
FAB	15.8 ± 2.4	15.9 ± 2.4	15.7 ± 2.5	0.321
FBI score	4.3 ± 6.2	2.4 ± 4.0	8.9 ± 7.9	<0.001*
ACE-R total score	78.1 ± 13.8	78.3 ± 13.9	77.6 ± 13.6	0.519
Attention/orientation	16.8 ± 1.6	16.8 ± 1.7	16.8 ± 1.5	0.860
Memory	20.7 ± 4.8	20.8 ± 4.8	20.4 ± 5.0	0.347
Verbal fluency	8.8 ± 2.7	8.9 ± 2.7	8.6 ± 2.6	0.097
Language	18.9 ± 4.9	18.8 ± 5.0	19.0 ± 4.9	0.615
Visuospatial ability	13.0 ± 3.3	13.1 ± 3.3	12.9 ± 3.2	0.317
ACE-R <75 (%)	33.8	33.8	33.8	0.991

ALS, amyotrophic lateral sclerosis; HDRS, hamilton depression rating scale; HARS, hamilton anxiety rating scale; FAB, frontal assessment battery; FBI, frontal behavioural inventory; ACE-R, addenbrooke’s cognitive examination-revised. *Significant difference. Student’s *t*-test and Chi-square test were used for between-group comparison. *p*-value represented the “*p*-value for between-group differences.”

For the health related QoL analysis, 642 patients and caregivers with complete available data were included (Table 3). Patients with apathy had higher proportion of reported problems in the anxiety/depression (68.5% vs. 51.8%, *p* < 0.001) dimensions. Furthermore, higher BDI and ZBI scores were found in caregivers in the apathy subgroup.

**Table 3:** Health-related quality of life of ALS patients and caregivers.

Variables	Total sample	Without apathy	With apathy	*p*-value
Number	642	423	219	
Health utility score	0.68 ± 0.25	0.70 ± 0.24	0.64 ± 0.27	0.009*
Mobility (problems, %)	56.3	61.2	57.9	0.231
Self-care (problems, %)	73.8	73.5	73.7	0.947
Usual activities (problems, %)	80.1	83.1	81.2	0.363
Pain/discomfort (problems, %)	45.6	42.8	51.1	0.044
Anxiety/ depression (%)	57.5	51.8	68.5	<0.001*
VAS score (Median)	64.2 ± 18.7	65.6 ± 18.5	61.6 ± 18.8	0.011*
BADL score	87.1 ± 16.0	88.1 ± 15.4	85.2 ± 17.1	0.029
IADL score	5.7 ± 2.4	5.8 ± 2.3	5.3 ± 2.5	0.008*
BDI for caregivers	2.9 ± 4.3	2.3 ± 3.9	4.0 ± 4.8	<0.001*
Zarit score	40.5 ± 13.2	38.6 ± 12.5	44.3 ± 13.8	0.001*

ALS, amyotrophic lateral sclerosis; VAS, visual analog scale; BADL, basic activities of daily living; IADL, instrumental activities of daily living; BDI, beck depression inventory. *Significant difference. Student’s *t*-test and Chi-square test were used for between-group comparison. *p*-value represented the “*p*-value for between-group differences.”

Logistic regression model showed that the onset region (OR = 0.789, 95%CI = 0.639–0.974, *p* = 0.027), ALSFRS-R score (OR = 0.0.965, 95%CI = 0.940–0.990, *p* = 0.007), depression (OR = 1.824, 95%CI = 1.289–2.580, *p* = 0.001), and anxiety (OR = 1.903, 95%CI = 1.359–2.664, p < 0.001) were associated with apathy (Table 4). Other parameters, including age, sex, marital status, BMI, family history, disease duration, diagnostic delay, stages, classical phenotype, disease progression rate, or ACE-R score showed no significant associations with apathy.

**Table 4:** Logistic regression model analyzed the factors associated with apathy in ALS patients.

Variable	OR	95% CI	*p*-value
Education	1.037	0.999–1.077	0.059
Onset region	0.789	0.639–0.974	0.027*
ALSFRS-R score	0.965	0.940–0.990	0.007*
Depression	1.824	1.289–2.580	0.001*
Anxiety	1.903	1.359–2.664	<0.001*

ALS, amyotrophic lateral sclerosis; ALSFRS-R, ALS functional rating scale revised; OR, odds ratios; CI, confidence intervals. *Significant difference.

**Figure 1:**
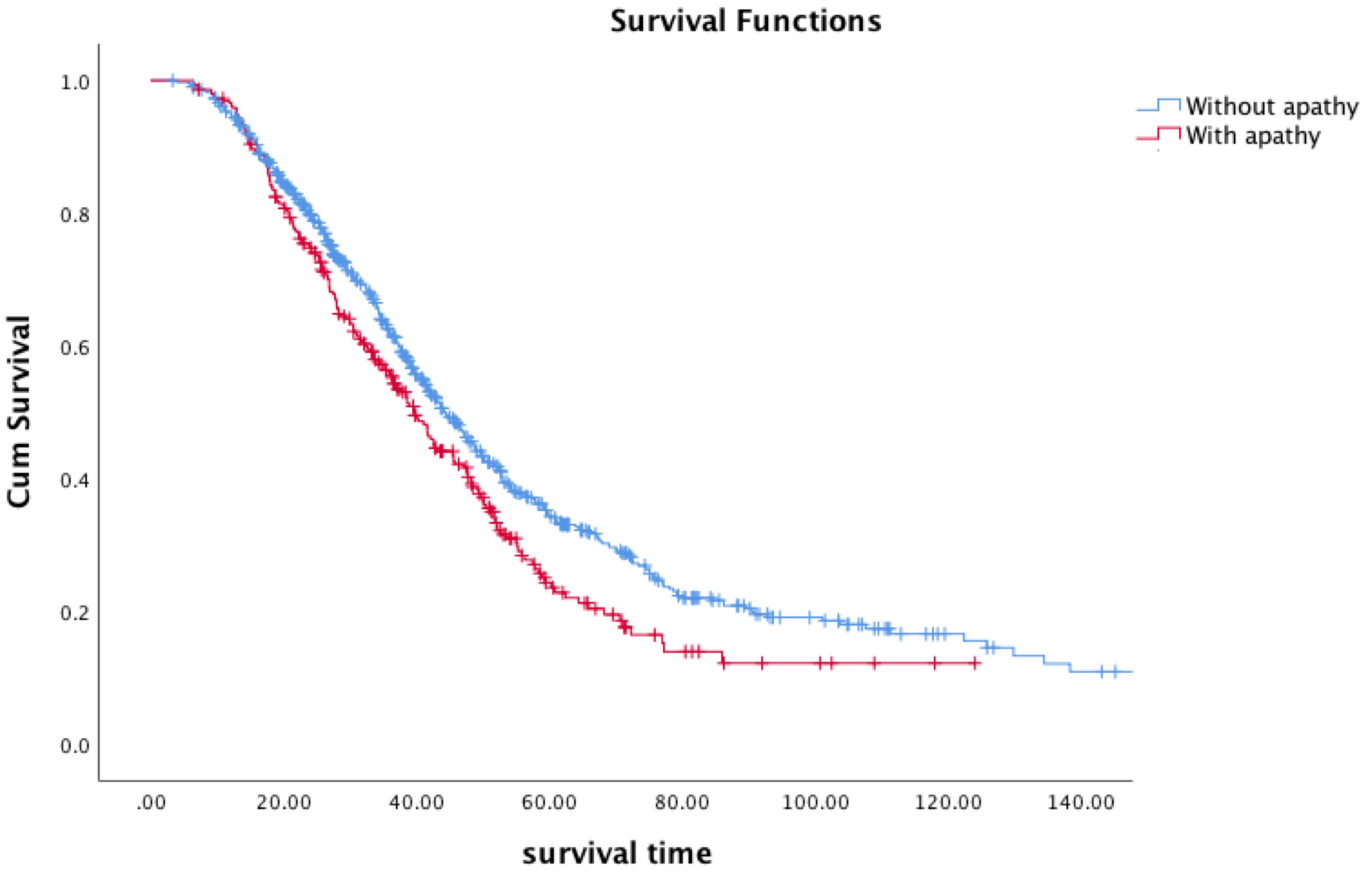
Survival curves according to apathy in ALS patients (blue, without apathy; red, with apathy).

In the end of the follow-up (April 2022), 641 patients (63.3%) had died, 346 patients (34.2%) were alive, and 26 patients (2.6) were lost to follow-up. The median survival time of all the patients was 42.8 months. 54.9% of the patients took riluzole. Kaplan–Meier survival analysis of all patients showed that patients with apathy had a shorter survival time than patients without survival (log-rank *p* = 0.006) (estimated median survival time:39.6 months vs. 44.4 months, Figure 1). Apathy had a significant effect on survival (HR = 1.210, 95%CI:1.016–1.440, *p* = 0.032) after adjusting for age, sex, marital status, BMI, family history, disease duration, onset region, classical phenotype, disease stage, ALSFRS-R score, depression, anxiety, ACER, and treatment. Using the apathy score instead of apathy groups, we generated similar findings for the apathy score (HR = 1.164. 95%CI:1.037–1.308, *p* = 0.010).

## Discussion

Overall, apathy (28.9%) is relatively frequent in Chinese ALS patients. Late stages of ALS patients had A higher prevalence of apathy. Furthermore, we showed that patient with apathy had lower health-related QoL and their caregivers had a higher care burden. We also highlighted that emotional state had a possible relationship with apathy in ALS. Apathy is strongly and independently associated with survival in ALS patients.

Apathy is a common neuropsychiatric symptom that has been observed in many neurodegenerative diseases. According to previous studies, the prevalence of apathy in ALS varies greatly, ranged from 8.6 to 80% (Santangelo et al., 2017). This large heterogeneity is likely associated with the array of methodological issues and assessment scales utilized to date. For example, in some, apathy was measured using non-ALS-specific tools, which might have led to bias in the results caused by muscle weakness or dysarthria, potentially contribute to an overestimation of apathy. To avoid the confounding influences of motor impairments, some studies have used the Dimensional Apathy Scale to evaluate apathy and apathetic subtypes in ALS patients. This scale provides scores both for patient-related and informant/caregiver-related apathy, and further comment on methodological bias for apathy in ALS when only caregivers are assessed. In a previous review, the prevalence of apathy was higher in studies that assessed by informant-rated tools (34%) than self-rated tools (25%) (Kutlubaev et al., 2022). In our study, based on information gathered from their caregivers, we found that apathy occurred in 28.9% of our patients. This finding is consistent with that of the prior review (Kutlubaev et al., 2022). Despite the methodological differences and bias on the prevalence of apathy, it appeared that specific neuroanatomical regions contributed to the observed apathy and apathy subtypes. Neuroimaging studies provided evidence of the involvement of brain circuits in apathy in ALS. A previous study observed that the severity of apathy was related to fractional anisotropy values in more widespread white matter areas, including frontal, parietal, and temporal lobes (Femiano et al., 2018). Another study found that increased initiation apathy correlated with reduced gray matter within the bilateral superior frontal gyrus and increased emotional apathy correlated with reduced gray matter in the prefrontal cortices and right anterior cingulate (Caga et al., 2021). Neuroimaging findings complement and extend the pathophysiological mechanisms of apathy in ALS.

The correlations between disease stage or severity and the frequency of apathy have inconsistent in prior reports. In one study, no significant difference was revealed in apathy in different stages according to King’s college staging system (Devenney et al., 2021). In addition, the severity of apathy was negative according to the ALSFRS-R score (Devenney et al., 2021). Other studies found that comparison of ALSFRS-R scores between apathetic and non-apathetic patients is controversial (Chiò et al., 2010; Caga et al., 2016; Santangelo et al., 2017; Salas et al., 2020). Another study indicated that behavioral changes were common and severe in advanced disease stages (Crockford et al., 2018). We found a higher prevalence of apathy in the late stages of ALS. In the multiple logistic regression model, ALSFRS-R scores other than disease stage were associated with apathy. Further longitudinal studies are required to explore the complex relationship in apathy and other ALS disease factors. For example, we found that the emergence of apathy in ALS was related to bulbar onset, which is reported in previous studies (Grossman et al., 2007; Chiò et al., 2010; Santangelo et al., 2017). This further supports the concept that bulbar onset is a risk factor for cognitive and behavioral involvement in ALS (Consonni et al., 2021; Yang et al., 2021).

For the effect of apathy on QoL, some studies have identified that increased severity of apathy was associated with lower patient QoL (Caga et al., 2018a), while other studies have found no such relationships (Chiò et al., 2010; Bock et al., 2016). In our study, patients with apathy had lower health-related QoL according to the EQ-5D-5L, which was most pronounced in anxiety/depression domains. Apathy may be a correlated factor for poor QoL in ALS, leading to issues with treatment compliance. Apathy also may impact different aspects of QoL, further highlighting the importance of assessing QoL domains other than those related to physical function in ALS with apathy (Pagnini, 2013). Early diagnosis and management of apathy would improve QoL in patients with ALS. We found that apathy in ALS was related to higher caregiver burden and depression. Previous study found that caregiver depression was correlated with patients’ apathy scores (Chiò et al., 2010). Another observational study also found that apathy aggravated the burden on caregivers (Caga et al., 2018a). Thus, the management of apathy and provision of tailored therapeutic interventions could help improve caregivers’ QoL.

In line with the previous study, we observed no significant difference for cognitive function in the subgroups with and without apathy (Caga et al., 2018b). They found that the difference in cognitive functioning using Mini-Addenbrooke’s Cognitive Examination between the groups was not statistically significant (Caga et al., 2018b). Another study found that patients with moderate to severe apathy had a higher percentage of cognitive impairment, as evaluated by the ACE-R (Caga et al., 2016). Other studies have also reported that patients with apathy perform worse in cognitive tests, such as verbal fluency, block design, and animal fluency (Grossman et al., 2007; Witgert et al., 2010). Furthermore, the association between the apathetic subtypes and cognitive function was explored. Initiation apathy was associated with verbal fluency deficits, while emotional apathy was associated with emotional recognition deficits, indicating the possible underlying pathological mechanisms of these cognitive and behavioral symptoms (Radakovic et al., 2017). The complex association between apathy and cognitive function is most likely due to methodological variability in assessing cognitive impartments. Therefore, future studies using ALS-specific instruments need to be performed to further clarify the association between apathy and cognitive function.

Few studies have so far reported on the relationship between apathy and emotional states. Patients with severe apathy have anxiety or depression than those with mild or moderate apathy (Witgert et al., 2010; Caga et al., 2018b; Siciliano et al., 2019). Previous study detected higher depression scores in ALS with apathy (Crockford et al., 2018). This significant correlation reflected an overlap between apathy and affective symptoms. In addition, a history of mood disorders increases the possibility of developing apathy in ALS patients (McHutchison et al., 2020). We also highlighted the possible relationship between emotional state and apathy in ALS using multivariate regression analysis. Patients with apathy had significantly higher HDRS and HARS scores than those without apathy. However, no association was found in other studies. There was no significant association between the level of apathy and depression and demoralization, suggesting that apathy and specific symptoms of depression may occur independently of each other in ALS. The association between apathy and emotional state is controversial in ALS, and further reports with stratification analysis are required to explore the possible relationships between these phenomena in ALS.

Apathy patients had shorter survival time in the Kaplan–Meier survival analysis compared to those without (estimated median survival time: 39.6 months vs. 44.4 months). Furthermore, apathy was found to have a negative effect on survival after adjusting for other parameters in our study, which is consistent with previous studies (Unglik et al., 2018). Higher level of apathy was significantly associated with mortality after controlling for clinical factors; for each one-unit change in the level of apathy, the risk of death more than tripled (Caga et al., 2016). Other studies have also indicated that apathy is a significant predictor of survival after when adjusted cognitive status and other clinical factors (Unglik et al., 2018). The presence of apathy preceded motor symptoms and worsen ALS prognosis. This might be due to poorer management with multidisciplinary care, that was associated with prolonged survival and better QoL. Early diagnosis and personalised intervention for apathy were helpful at reducing the involvement in worse ALS outcomes.

The inclusion of non-specific apathy scale is a major limitation of the study. Apathy was evaluated based on one item from FBI questionnaire. In addition, using a single item to categorize participants into two subgroups may result in further methodological bias. Another limitation of the study is the use of a less sensitive and specific scale for cognitive impairment in ALS. Previous studies have shown that ACE may not captured mild cognitive impairment in ALS and has higher ceiling effects compared to ALS-specific scales (De Icaza Valenzuela et al., 2018; Kourtesis et al., 2020). ALS-specific and apathy-specific scales were needed to evaluate cognition and apathy in ALS. Finally, other factors, such as the *C9orf72* gene mutation, which interferes with cognitive function, should been considered in the Cox analysis (Table 5).

**Table 5:** Multivariate Cox analyses of factors associated with survival in ALS patients.

Variable	HR (95% CI)	*p*-value
Age	1.018 (1.011–1.025)	<0.001*
Sex	0.743 (0.627–0.880)	0.001*
Family history	2.189 (1.188–4.031)	0.012*
Onset region	0.624 (0.504–0.771)	<0.001*
Disease stages	1.223 (1.108–1.350)	<0.001*
ALSFRS-R score	0.932 (0.916–0.948)	<0.001*
Disease duration	0.914 (0.904–0.925)	<0.001*
Using riluzole	0.491 (0.419–0.576)	<0.001*
PEG	0.492 (0.349–0.695)	<0.001*
Apathy	1.210 (1.016–1.440)	0.032*

ALS, amyotrophic lateral sclerosis; HR, hazard ratios; CI, confidence intervals; ALSFRS-R, ALS functional rating scale revised; PEG, percutaneous endoscopic gastrostomy. *Significant difference.

## Conclusion

Apathy is common in Chinese ALS patients and has a negative effect on survival. Apathy in ALS is related not only to the severity of the disease, but also to some non-motor symptoms, such as depression and anxiety disorders. Apathy is an independent prognostic factor for ALS. Early intervention and management of apathy could prolong survival and improve QoL in patients with ALS.

## Data availability statement

The raw data supporting the conclusions of this article will be made available by the authors, without undue reservation.

## Ethics statement

The studies involving human participants were reviewed and approved by the Ethics Committee of West China Hospital of Sichuan University. The patients/participants provided their written informed consent to participate in this study.

## Author contributions

Q-QW research project (conception, organization, execution), statistical analysis (design), and manuscript (writing of the first draft). YG statistical analysis (review and critique) and patients enrollment. SL and TY patients enrollment and follow up. YH, RO, JL, and QJ patients enrollment. HS research project (conception), statistical analysis (review and critique), and manuscript (review and critique). All authors read and approved the final manuscript.

## Funding

This study was supported by funding from the National Natural Science Foundation of China (Grant No. 82101485), Sichuan Science and Technology Program (Grant No. 2022ZDZX0023), Science and Technology Commission Foundation of Chengdu City (Grant No. 2021-YF05-00242-SN), and Postdoctoral Science Foundation of Sichuan University (Grant No. 2021SCU12019).

## Conflict of interest

The authors declare that the research was conducted in the absence of any commercial or financial relationships that could be construed as a potential conflict of interest.

## Publisher’s note

All claims expressed in this article are solely those of the authors and do not necessarily represent those of their affiliated organizations, or those of the publisher, the editors and the reviewers. Any product that may be evaluated in this article, or claim that may be made by its manufacturer, is not guaranteed or endorsed by the publisher.
